# Insights into the Cytotoxicity and Irritant Potential of Chlorhexidine Digluconate: An In Vitro and In Ovo Safety Screening

**DOI:** 10.3390/dj12070221

**Published:** 2024-07-17

**Authors:** Stefania Dinu, Anamaria Matichescu, Roxana Buzatu, Iasmina Marcovici, Andreea Geamantan-Sirbu, Alexandra Denisa Semenescu, Remus Christian Bratu, Dana-Cristina Bratu

**Affiliations:** 1 Department of Pedodontics, Faculty of Dental Medicine, “Victor Babeș” University of Medicine and Pharmacy, No. 9, Revolutiei 1989 Bv., 300041 Timisoara, Romania; dinu.stefania@umft.ro; 2 Pediatric Dentistry Research Center, Faculty of Dental Medicine, Victor Babeș University of Medicine and Pharmacy, No. 9, Revolutiei Bv., 300041 Timisoara, Romania; 3 Department of Preventive, Community Dentistry and Oral Health, Translational and Experimental Clinical Research Centre in Oral Health, University of Medicine and Pharmacy “Victor Babeș”, 300040 Timisoara, Romania; 4 Translational and Experimental Clinical Research Centre in Oral Health, “Victor Babeș” University of Medicine and Pharmacy, 14A Tudor Vladimirescu Ave., 300173 Timișoara, Romania; 5 Department of Dental Aesthetics, Faculty of Dental Medicine, “Victor Babeș” University of Medicine and Pharmacy Timisoara, Revolutiei Boulevard 9, 300041 Timisoara, Romania; roxana.buzatu@umft.ro; 6 Faculty of Pharmacy, “Victor Babes” University of Medicine and Pharmacy Timisoara, Eftimie Murgu Square No. 2, 300041 Timisoara, Romania; iasmina.marcovici@umft.ro (I.M.); andreea.geamantan@umft.ro (A.G.-S.); alexandra.scurtu@umft.ro (A.D.S.); 7 Research Center for Pharmaco-Toxicological Evaluations, Faculty of Pharmacy, “Victor Babeș” University of Medicine and Pharmacy Timisoara, Eftimie Murgu Square No. 2, 300041 Timisoara, Romania; 8 Faculty of Dental Medicine, “Victor Babeș” University of Medicine and Pharmacy Timișoara, 2 Eftimie Murgu Square, 300041 Timișoara, Romania; remus.bratu@student.umft.ro; 9 Department of Orthodontics II, Faculty of Dental Medicine, “Victor Babeș” University of Medicine and Pharmacy Timișoara, 2 Eftimie Murgu Square, 300041 Timișoara, Romania; bratu.cristina@umft.ro

**Keywords:** chlorhexidine digluconate, cutaneous cells, cytotoxicity, dentistry, gingival fibroblasts, irritant potential, safety screening

## Abstract

Chlorhexidine (CHX) represents one of the most commonly used antiseptics in dentistry and other medical–pharmaceutical fields due to its broad-spectrum antimicrobial activity. However, the potential toxic events arising from its common use in practice has become a subject of increasing concern. Thus, the present study was designed to investigate the potential toxicity of CHX digluconate at concentrations covering its antibacterial properties (0.0002–0.2%) in HGF primary gingival fibroblasts, HaCaT immortalized human keratinocytes, and JB6 Cl 41-5a epidermal cells, as well as its irritant action in ovo. Our results indicate that CHX exerted a concentration- and time-dependent cytotoxicity in all cell lines, which was evidenced by the reduction in cell viability, number, and confluence, damaged cell membrane integrity, impaired cell morphology, and specific apoptotic nuclear shape. The highest cytotoxicity was caused by CHX digluconate 0.02% and 0.2%, concentrations, at which an irritant effect on the chorioallantoic membrane was also observed. The novel findings revealed in this research contribute to the overall safety profile of CHX and stand as a basis for further investigations in this regard.

## 1. Introduction

Chlorhexidine (CHX) is a bisbiguanide antiseptic molecule that is composed of two biguanide units which are interconnected by a central hexamethylene bridge and which can currently be found in different forms such as digluconate, acetate, or hydrochloride salts [1,2,3]. It was discovered in the 1950s and has been broadly used ever since in various medical–pharmaceutical fields [1]. CHX remains a gold-standard antimicrobial substance in dental practice, with applications in general dentistry as well as endodontics, periodontology, and surgery [2,4]. Formulated as gels, varnishes, solutions, mouthwashes, aerosols, sprays, and disks, CHX has been efficiently applied as an adjuvant in mechanical oral hygiene, dental carries management, plaque control, gingival inflammation, and bleeding reduction [2,5]. It is also applied to various medical devices such as vascular catheters, antibacterial dressings, or dental implants, reportedly due to its higher efficacy in reducing surgical infections compared to povidone–iodine [6,7].

CHX possesses a broad antimicrobial spectrum of action, with demonstrated activity against Gram-positive and Gram-negative bacteria, viruses, and yeasts [8]. The CHX mechanism of action derives from its ability to penetrate and disrupt the bacterial cell wall, precipitate cytoplasmic contents, cause cytoplasmic membrane damage and integrity loss, outflow low-molecular-weight cytoplasmic constituents (e.g., potassium ions), and inhibit enzymatic activity [7,8,9]. Nonetheless, its antibacterial activity is dose-dependent: at low concentrations, CHX exerts a bacteriostatic effect, while at high concentrations, it acts as a bactericidal agent [3,8]. CHX has cationic loading properties resulting from the single chlorine atoms linked to both phenol rings [9], thus efficiently binding to anionic proteins which leads to a prolonged antiseptic effect [6]. CHX can also adsorb to various surfaces within the oral cavity, such as the mucosa and the pellicle-coated teeth, thus providing a sustained antimicrobial activity that restricts bacterial growth for at least 24 h; however, it also causes retention, with higher CHX concentrations and longer time exposures being associated with an increase in its retention within the oral mucosa [10].

The widespread use of CHX-based formulations raised several concerns regarding its safety, with this antiseptic being currently associated with toxic events that range from numbness, taste loss, xerostomia, and allergic reactions to life-threatening anaphylaxis [2]. The most serious adverse effects associated with the oral use of CHX remain the occurrence of type IV and type I hypersensitivity reactions, the reported incidence rates being 0.78 per 100,000 cases [2]. CHX-caused adverse reactions require a comprehensive investigation to reduce their occurrence and ensure patient safety by optimizing the used concentrations and improving the clinical protocol; currently, the safest and optimal application regimes are still unclear [11]. Therefore, previous studies have addressed the potential toxicity of CHX. For instance, its cytotoxic effects immediately after the exposure of several healthy cell types (e.g., gingival and dermal fibroblasts, osteoblasts, etc.) to CHX were disclosed [12,13,14]. However, despite the previous evidence on the cytotoxicity of CHX, its long-lasting effects, and retention within the oral cavity, the delayed cytopathic effects of CHX remain incompletely explored at present. Indeed, a recent report demonstrated the deleterious effects of CHX gluconate in fibroblasts, myoblasts, and osteoblasts at 48 h after the cells’ treatment [15]. Nonetheless, to complete these previous findings on the delayed toxic properties of CHX in healthy cells, the current study aimed to provide a glance at the potential in vitro toxicity of CHX digluconate in HGF primary gingival fibroblasts, HaCaT immortalized human keratinocytes, and JB6 Cl 41-5a epidermal cells at 24 h after their exposure to CHX 0.0002%, 0.002%, 0.02%, and 0.2% for short-term intervals. These experiments were conducted with the consideration that these panels of cell lines cover the current indications of CHX—which is used as an antiseptic in dental and cutaneous applications [2,7]—as well as accounting for the fact that the selected concentrations are clinically relevant for its bacteriostatic and bactericidal properties [2] and that CHX presents with tissue retention, being detected in the oral cavity even at 24 h after application [16,17,18]. Furthermore, taking into account the direct contact of CHX with both the cutaneous surface and the mucosa of the oral cavity during clinical use, the potential in ovo irritant property of CHX was also determined using the Hen’s Egg-Chorioallantoic Membrane (HET-CAM) test due to its wide application in the assessment of the irritant effects on mucous membranes, and its correlation with the results of dermal irritation tests [19,20].

## 2. Materials and Methods

### 2.1. Reagents and Equipment

Chlorhexidine digluconate solution 2% (Gluco Chex-In 2%) was obtained from CERKAMED (Stalowa Wola, Polonia). Dulbecco’s Modified Eagle Medium (DMEM- 30-2002 ™), Fibroblast Basal Medium (ATCC PCS-201), Fibroblast Growth Kit-Low serum (ATCC PCS-201-04), penicillin/streptomycin/amphotericin B (PCS-999-002™), dimethylsulfoxide (DMSO, 4-X™), Eagle Minimum Essential Medium (30-2003 ™), fetal bovine serum (FBS- 30-2020 ™), a penicillin/streptomycin mixture, and trypsin-EDTA solution were purchased from American Type Culture Collection (ATCC) Manassas, VA, USA. The phosphate-buffered saline (PBS), the MTT (3-(4,5-dimethylthiazol2-yl)-2,5-diphenyltetrazolium bromide) viability kit, and Triton X-100 were purchased from Sigma-Aldrich, Merck KGaA (Darmstadt, Germany). The lactate dehydrogenase (LDH) cytotoxicity kit, Hoechst 33342 dye, and TexasRed™-X Phalloidin were procured from ThermoFisher Scientific (Waltham, MA, USA). Bovine serum albumin (BSA) was obtained from Cell Signaling Technology (Danvers, MA, USA). Paraformaldehyde 4% was delivered by Santa Cruz Biotechnology (Dallas, TX, USA). Cytation 5 (plate reader), and Lionheart FX (automated microscope) were provided by BioTek Instruments Inc. (Winooski, VT, USA), while the SteREO Discovery.V8 stereomicroscope was obtained from ZEISS (Jena, Germany).

### 2.2. Computational Assessment of CHX in Terms of Drug Likeness and Potential Toxicity

For the initial computational description of the compound of interest, CHX, in terms of potential drug-like nature and toxicity, the OSIRIS Property Explorer was employed as previously reported by Dehelean et al. [21]. The canonical SMILES (simplified molecular-input line-entry system) for CHX was obtained from Pubchem [22].

### 2.3. Cell Culture Conditions

HGF primary gingival fibroblasts (PCS-201-018™; ATCC, Manassas, VA, USA), Ha-CaT immortalized human keratinocytes (300493; CLS, Eppelheim, Germany), and JB6 Cl 41-5a neonatal BALB/c epidermal cells (CRL-2010™; ATCC, Manassas, VA, USA) were used in this study. Each cell line was grown in its specific culture medium, as follows: HGF cells were cultured in fibroblast growth medium supplemented with Fibroblast Growth Kit-Low serum and penicillin/streptomycin/amphotericin B (PCS-999-002™); HaCaT cells were grown in Dulbecco’s Modified Eagle medium supplemented with 10% fetal bovine serum and a 1% mixture of penicillin/streptomycin; JB6 Cl 41-5a was cultured in Eagle Minimum Essential Medium supplemented with 5% fetal bovine serum and a 1% penicillin/streptomycin antibiotic mixture. Cells were maintained throughout the experiments under standard conditions of 37 °C and 5% CO_2_.

### 2.4. Applied Treatment Regimen

The four tested samples (CHX 0.0002%, CHX 0.002%, CHX 0.02%, and CHX 0.2%) were obtained by diluting the 2% CHX stock solution. The cells (HGF, HaCaT, and JB6 Cl 41-5a) were treated with test samples for 1, 2, and 3 min, respectively, washed with PBS to remove CHX traces, and incubated for 24 h. All the experiments were performed at the end of the incubation time.

### 2.5. Cell Viability Assay

Cell viability was analyzed using the MTT (3-(4,5-dimethylthiazol-2-yl)-2,5-diphenyltetrazolium bromide) test. Shortly, at the end of the treatment time, 100 µL of fresh medium and 10 µL of MTT were added to each well and the plate was placed in the incubator. After 3 h of incubation, 100 µL of MTT solubilizing solution were added, the plate was maintained for 30 min at room temperature, and the absorbance was read at 570 and 630 nm wavelengths using Cytation 5.

### 2.6. Assessment of Cell Confluence and Cell Number

To assess the influence of CHX treatment on HGF, HaCaT, and JB6 Cl 41-5a cells’ confluence and number, representative images were taken using the objective 4× (PL FL), on the Lionheart FX automated microscope equipped with a wide-field-of-view (WFOV) camera and a high-contrast brightfield. Each image was further automatically analyzed using the Cell Analysis Tool provided by the Gen5™ Microplate Data Collection and Analysis Software (Version 3.14) from BioTek Instruments Inc. (Winooski, VT, USA) by following the procedure for measuring confluence using the high-contrast brightfield available on the manufacturer’s site [23].

### 2.7. Evaluation of Lactate Dehydrogenase (LDH) Leakage

The ability of CHX (0.0002%, 0.002%, 0.02%, and 0.2%) to damage cell membranes was evaluated by quantifying the release of the cytosolic LDH in the culture medium after treatment. In this sense, 50 µL of culture medium (containing released LDH) was transferred into 96-well culture plates and mixed with 50 µL of the reaction mixture. The plates were then incubated for 30 min, and after this period of time, 50 µL of the stop solution was added to each well. Subsequently, the absorbance was read at 490 nm and 680 nm using Cytation 5.

### 2.8. Bright-Field Cell Morphology Assessment

The impact of CHX (0.0002%, 0.002%, 0.02%, and 0.2%) on HGF, HaCaT, and JB6 Cl 41-5a cell morphologies was investigated by taking representative images of the control and treated cells under brightfield illumination (at magnification 20×) on the Lionheart FX automated microscope; the obtained pictures were processed in the Gen5™ Microplate Data Collection and Analysis Software (Version 3.14) from BioTek Instruments Inc. (Winooski, VT, USA).

### 2.9. TexasRed™-X Phalloidin Staining

The visualization of cytoskeletal F-actin was performed using the TexasRed™-X Phalloidin dye. Simply, HGF, HaCaT, and JB6 Cl 41-5a cells were grown in 96-well black plates with a clear bottom, left to attach, and exposed to the CHX treatment regimen. Next, the cells were treated with paraformaldehyde 4% (fixation solution), Triton-X 0.1% (permeabilization solution), and BSA 1% (blocking solution), at room temperature, for 15 min each. Finally, the cells were treated for 30 min at room temperature with TexasRed™-X Phalloidin dye diluted in BSA 0.1%, washed several times with PBS, and imaged using the Lionheart FX automated microscope and the Gen5™ Microplate Data Collection and Analysis Software, BioTek Instruments Inc. (Winooski, VT, USA). The nuclei were also stained with DAPI for 5 min at room temperature.

### 2.10. Hoechst 33342 Nuclear Staining

A Hoechst 33342 staining was performed to underline the changes induced by CHX on the nuclear shape of HGF, HaCaT, and JB6 Cl 41-5a cells. Briefly, after the applied treatment, the Hoechst staining solution was prepared (dilution 1:2000 in PBS), the culture medium from the plates was removed, the prepared staining solution was added, and the plates were incubated in a dark place for 5–10 min. After this time, the staining solution was removed, and the cells were washed three times with PBS. The processing and analysis of the images were performed using the Lionheart FX automated microscope and the Gen5™ Microplate Data Collection and Analysis Software (Version 3.14) from BioTek Instruments Inc. (Winooski, VT, USA).

Based on the following formula [24], the apoptotic index (AI) was calculated for each cell line:

$$AI(\%)=\frac{Number\;of\;apoptotic\;cells}{Total\;number\;of\;cells}\;\times \;100$$

### 2.11. The HET-CAM Assay

To investigate the irritant potential of the antiseptic CHX, the HET-CAM method was applied. The following steps were applied to perform this test: (i) the eggs were disinfected with alcohol (70% *v*/*v* concentration), dated, and horizontally incubated at a controlled temperature of 37–37.5 °C; (ii) on the 4th day of incubation, the eggshell was perforated and a volume of approximately 5–7 mL of albumen was removed to enable the chorioallantoic membrane to detach from the upper part of the eggshell; then, the perforation was covered with adhesive tape; (iii) in the 5th day of incubation, a window was cut in the upper part of the egg to observe the vessels of the chorioallantoic membrane, and after the perforation was performed, the eggs were covered with adhesive tape and incubated until the testing began. During the entire experiment, the incubator was monitored daily to ensure the proper temperature and humidity. The concentrations of CHX chosen for this assay were 0.02% and 0.2%, which are the highest ones to be tested in vitro that have produced significant cytotoxicity. Sodium dodecyl sulfate (SDS) 1% was used as a positive control and distilled water (H_2_O) was used as a negative control. A measure of 600 µL of each sample (positive control, negative control, CHX 0.002%, and CHX 0.2%) was applied to the chorioallantoic membrane, and vascular lysis, coagulation, and hemorrhage were monitored for 5 min. Representative images of the membrane were taken before the application of the samples (at T0) and at the end of the treatment (T5). The images were carried out using the Discovery v.8 stereomicroscope and the ZEN core 3.8 software. The irritation score (IS) was determined using the formula presented below:

$$IS=5\times \frac{301-H}{300}+7\times \frac{301-L}{300}+9\times \frac{301-C}{300}$$

IS is a parameter that determines the irritant potential of compounds by measuring the time when changes (H—hemorrhage; L—vascular lysis; C—coagulation) occur at the vascular level. Based on its value, the test substance can be classified as non-irritant if IS = 0–0.9, irritant if IS = 1–8.9, and severely irritant when IS = 9–21 [25,26].

## 3. Results

### 3.1. Computational Predictions of CHX Drug Likeness and Toxic Risk

The drug likeness and toxicity of CHX were computationally predicted using the OSIRIS Property Explorer program. The results, indicated in Table 1, suggest that CHX presents a negative drug likeness, but an overall positive drug score and lacks mutagenic, tumorigenic, irritant, or reproductive toxicity potential.

### 3.2. CHX Impairs Cell Viability in a Concentration-Dependent Manner

**Figure 1:**
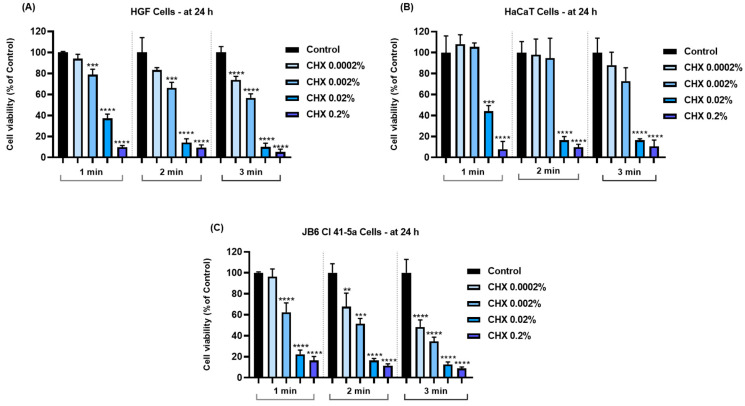
Graphical representation of cell viability percentages obtained 24 h after the 1, 2, and 3 min treatments of (**A**) HGF, (**B**) HaCaT, and (**C**) JB6 Cl 41-5a cells with CHX (0.0002%, 0.002%, 0.02%, and 0.2%). The results are presented as percentages (%), normalized to control (cells without CHX treatment). All data are expressed as mean values ± SD obtained from three independent experiments performed in triplicate. For analyzing the statistical differences between the control group and the CHX-stimulated groups, a one-way ANOVA test was conducted, followed by the Dunnet’s multiple comparison post-test; “*” indicates statistical significance (** *p* < 0.01; *** *p* < 0.001; **** *p* < 0.0001).

To investigate the impact of CHX (0.0002%, 0.002%, 0.02%, and 0.2%) on the viability of HGF, HaCaT, and JB6 Cl 41-5a cells, an MTT assay was performed 24 h after their exposure for 1, 2, and 3 min to CHX. The obtained results indicated a time- and concentration-dependent response to CHX cytotoxicity; the most significant viability reductions (<20%) were noticed at the highest tested concentrations—0.02% and 0.2%—and at the longest exposure time (3 min). Overall, a cell type-dependent sensitivity to CHX was also observed. The viability of HGF cells (Figure 1A) was lowered by all concentrations of CHX and at all time intervals; however, statistical significance was reached only for the concentrations of 0.002%, 0.02%, and 0.2% after 1 and 2 min of treatment, as well as for all tested concentrations after 3 min of exposure. HaCaT cells (Figure 1B) presented the highest resistance to the cytotoxic effects of CHX, their viability being significantly reduced by <50% only at concentrations of 0.02% and 0.2%, after 1, 2, and 3 min. Moreover, the 1 min treatment of HaCaT cells with CHX 0.0002% and 0.002% exerted a slight stimulatory effect on cell viability that reached values of 108.18% and 105.17%, respectively. Lastly, in the case of JB6 Cl 41-5a cells (Figure 1C), CHX lacked cytotoxicity only at the concentration of 0.0002% when tested for 1 min, while all the other concentrations and treatments significantly impaired their viability, which was reduced by <70%.

### 3.3. CHX Reduces Cell Number and Confluence in a Concentration-Dependent Manner

**Figure 2:**
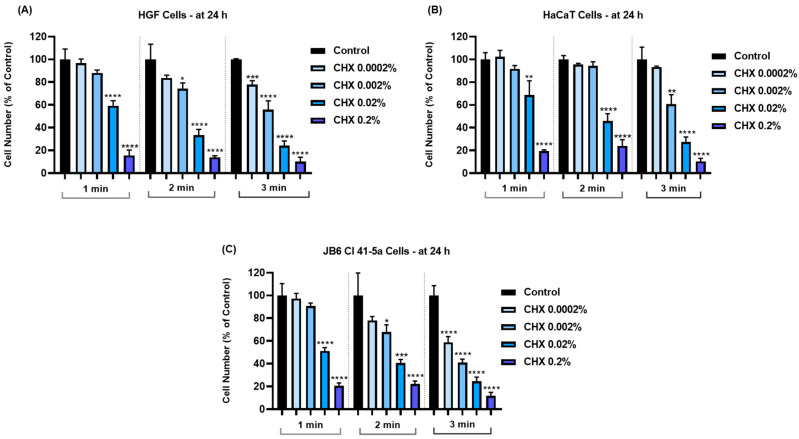
Graphical representation of cell number in (**A**) HGF, (**B**) HaCaT, and (**C**) JB6 Cl 41-5a cells at 24 h after their 1, 2, and 3 min treatments with CHX (0.0002%, 0.002%, 0.02%, and 0.2%). The results are presented as percentages (%) normalized to control (cells without CHX treatment). All data are expressed as mean values ± SD obtained from three independent experiments performed in triplicate. For analyzing the statistical differences between the control group and the CHX-stimulated groups, a one-way ANOVA test was conducted, followed by the Dunnet’s multiple comparison post-test; “*” indicates statistical significance * *p* < 0.05; ** *p* < 0.01; *** *p* < 0.001; **** *p* < 0.0001).

Next, the impact of CHX on cell number and confluence was assessed. As shown in Figure 2, the decrease in cell number was directly proportional to the increase in exposure interval and concentration. Thus, the most considerable reductions in cell number were induced by CHX 0.2%, after the 3 min treatment, in all cell lines.

**Figure 3:**
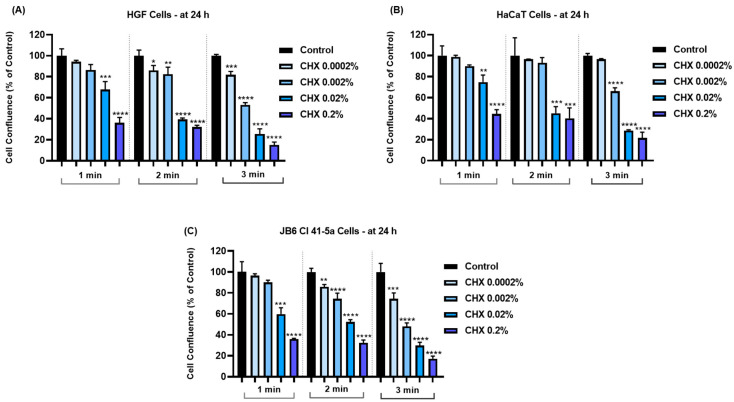
Graphical representation of cell confluence in (**A**) HGF, (**B**) HaCaT, and (**C**) JB6 Cl 41-5a cells at 24 h after their 1, 2, and 3 min treatments with CHX (0.0002%, 0.002%, 0.02%, and 0.2%). The results are presented as percentages (%) normalized to control (cells without CHX treatment). All data are expressed as mean values ± SD obtained from three independent experiments performed in triplicate. For analyzing the statistical differences between the control group and the CHX-stimulated groups, a one-way ANOVA test was conducted, followed by the Dunnet’s multiple comparison post-test; “*” indicates statistical significance (* *p* < 0.05; ** *p* < 0.01; *** *p* < 0.001; **** *p* < 0.0001).

Similarly, the confluence of all cell lines (Figure 3) was reduced in a concentration- and time-dependent manner; the highest loss was determined after their treatment with CHX at the highest concentration and longest exposure time.

### 3.4. CHX-Induced Cell Membrane Damage and Lactate Dehydrogenase Leakage

**Figure 4:**
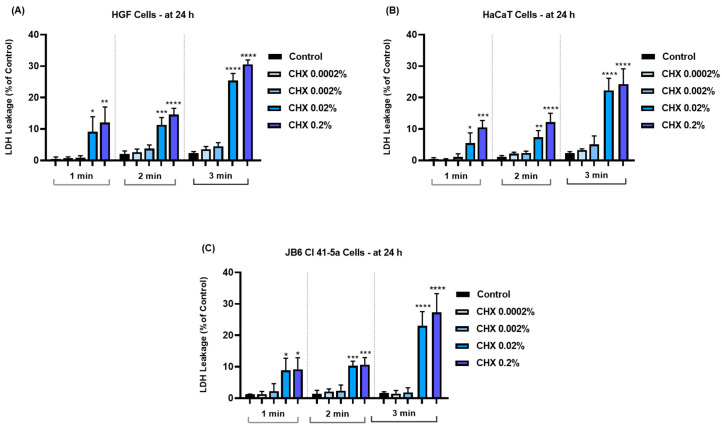
Graphical representation of lactate dehydrogenase (LDH) leakage in (**A**) HGF, (**B**) HaCaT, and (**C**) JB6 Cl 41-5a cells at 24 h after their 1, 2, and 3 min treatments with CHX (0.0002%, 0.002%, 0.02%, and 0.2%). The results are presented as percentages (%) normalized to control (cells without CHX treatment). All data are expressed as mean values ± SD obtained from three independent experiments performed in triplicate. For analyzing the statistical differences between the control group and the CHX-stimulated groups, a one-way ANOVA test was conducted, followed by the Dunnet’s multiple comparison post-test; “*” indicates statistical significance (* *p* < 0.05; ** *p* < 0.01; *** *p* < 0.001; **** *p* < 0.0001).

Further, the influence of CHX on cell membrane integrity and permeability was evaluated by measuring the LDH release in culture media. As indicated by the results from Figure 4, significant leakage was induced at all treatment times in the treated cell lines. However, this occurred only at the highest concentrations tested of 0.02% and 0.2%. The cytotoxic effect was also time-dependent; the highest amounts of LDH (>20% compared to <3% in the case of the respective Controls) were released in all cells exposed to CHX for 3 min.

### 3.5. CHX-Induced Changes in Cell Morphology

**Figure 5:**
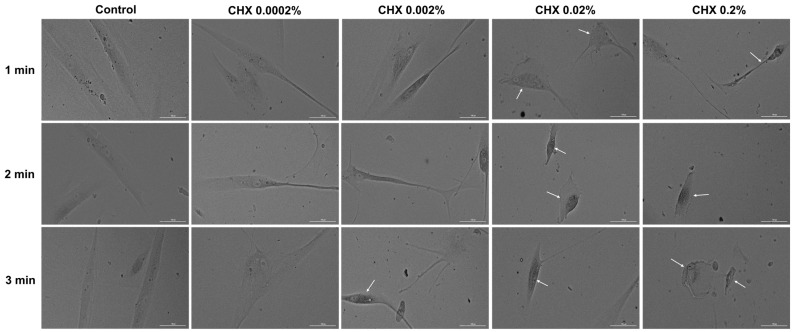
Representative images illustrating the morphological changes observed at 24 h after the 1, 2, and 3 min stimulations of HGF cells with CHX (0.0002%, 0.002%, 0.02%, and 0.2%). The pictures were taken at a magnification of 20×, and the scale bar indicates 100 μm. The white arrows indicate changes in cell morphology.

Considering the results obtained so far, the next step in the exploration of CHX cytotoxicity was the evaluation and analysis of HGF, HaCaT, and JB6 Cl 41-5a cells’ morphology 24 h after their 1, 2, and 3 min treatments with CHX. The results reinforced the fact that CHX causes a toxic effect that is mainly dependent on the selected cell line, exposure time, and concentration. In the case of HGF cells (Figure 5), CHX concentrations of 0.0002% and 0.002% induced cell elongation at all treatment times, while at higher concentrations, a reduction in the cells’ longitudinal axis can be observed. The massive shrinkage of the cells exposed to CHX 0.2% for 1, 2, and 3 min, as well as debris, are also visible.

**Figure 6:**
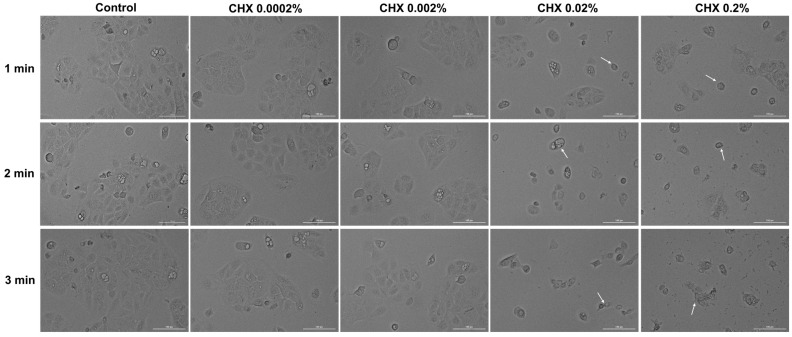
Representative images illustrating the morphological changes observed at 24 h after the 1, 2, and 3 min stimulations of HaCaT cells with CHX (0.0002%, 0.002%, 0.02%, and 0.2%). The pictures were taken at a magnification of 20×, and the scale bar indicates 100 μm. The white arrows indicate changes in cell morphology.

Regarding HaCaT cells (Figure 6), CHX 0.0002% and 0.002% did not induce noticeable changes in their shape, with the cells’ appearance being similar to that of the control. However, obvious morphological impairments can be observed starting from the concentration of 0.02% at all three exposure periods; this is highlighted by a reduction in cell confluence and colony number, as well as cell rounding and shrinkage. Besides these cytotoxic effects, at 0.02% CHX treatment led to the accumulation of debris resulting from cell death.

**Figure 7:**
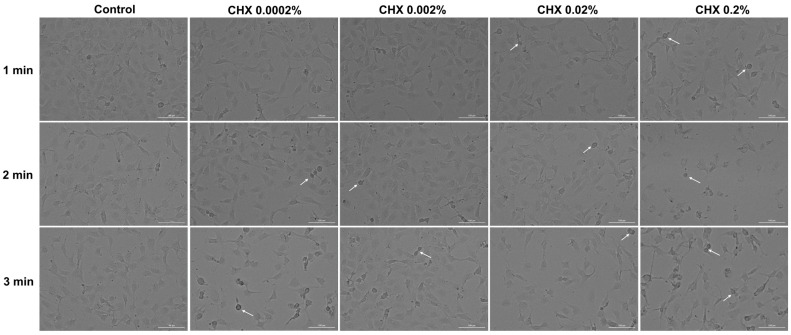
Representative images illustrating the morphological changes observed at 24 h after the 1, 2, and 3 min stimulations of JB6 Cl 41-5a cells with CHX (0.0002%, 0.002%, 0.02%, and 0.2%). The pictures were taken at a magnification of 20×, and the scale bar indicates 100 μm. The white arrows indicate changes in cell morphology.

In the JB6 Cl 41-5a cell line (Figure 7), changes in cell structure are evident even at the lowest CHX concentration (0.0002%) after 3 min of treatment. A decrease in confluency was also observed with increasing doses and stimulation time. Also, the rounding of the cells, shrinkage, and detachment from the plate can be observed at all concentrations after 3 min of exposure. The most significant impairment in morphology was caused by CHX 0.2%, applied for 1, 2, and 3 min.

### 3.6. CHX-Induced F-Actin Rearrangements

**Figure 8:**
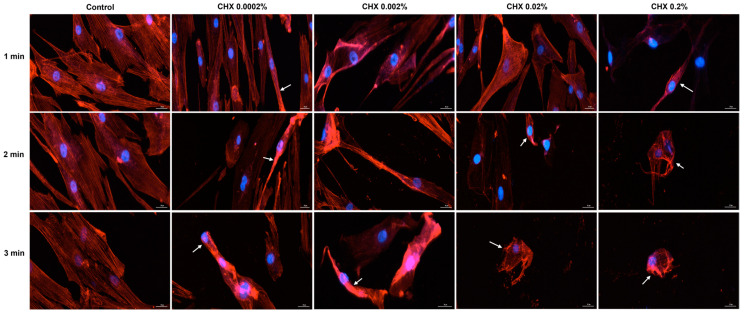
Representative images illustrating the distribution of cytoskeletal F-actin at 24 h after the 1, 2, and 3 min stimulations of HGF cells with CHX (0.0002%, 0.002%, 0.02%, and 0.2%). The pictures were taken at a magnification of 20×, and the scale bar indicates 30 μm. The white arrows indicate F-actin alterations.

Based on the cell morphology results, the study continued by evaluating the impact of CHX on the distribution of cytoskeletal F-actin within HGF, HaCaT, and JB6 Cl 41-5a cells at 24 h after their 1, 2, and 3 min treatments with CHX. Figure 8 depicts the F-actin aspect in CHX-treated HGF cells. As observed, all CHX concentrations triggered the constriction of F-actin bundles (white arrows); however, the cell aspect was dependent on the tested dose. At 0.0002%, CHX treatment enhanced the length of HGF cells, which became more elongated compared to the respective controls, while at higher concentrations, significant cell shrinkage and length reduction were observed.

**Figure 9:**
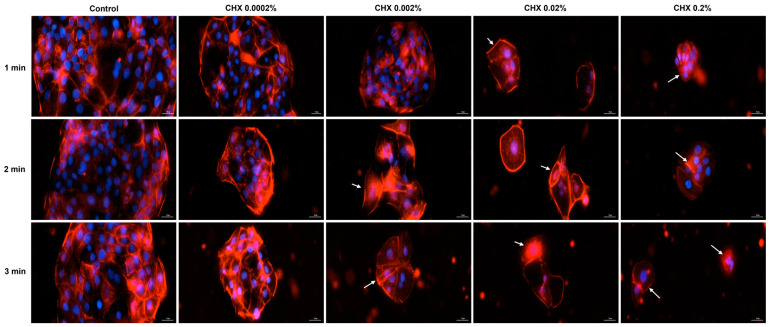
Representative images illustrating the distribution of cytoskeletal F-actin at 24 h after the 1, 2, and 3 min stimulations of HaCaT cells with CHX (0.0002%, 0.002%, 0.02%, and 0.2%). The pictures were taken at a magnification of 20×, and the scale bar indicates 30 μm. The white arrows indicate F-actin alterations.

Regarding the HaCaT cells (Figure 9), the most significant modifications in F-actin arrangement, and condensation of F-actin filaments were noticed at the highest concentrations tested (0.02% and 0.2%), at all treatment intervals. The alterations in F-actin distribution were accompanied by visible morphological changes, the cells becoming spherical and shrunken in shape.

**Figure 10:**
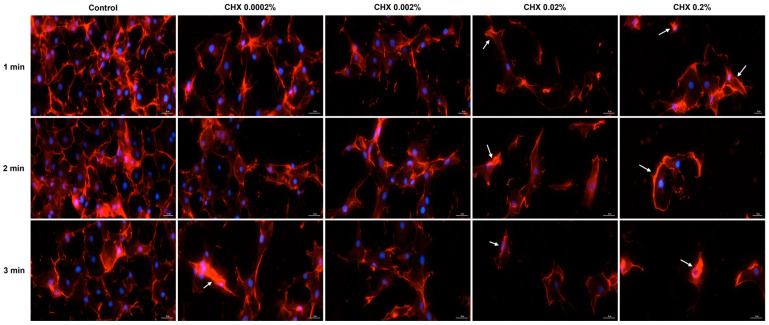
Representative images illustrating the distribution of cytoskeletal F-actin at 24 h after the 1, 2, and 3 min stimulations of JB6 Cl 41-5a cells with CHX (0.0002%, 0.002%, 0.02%, and 0.2%). The pictures were taken at a magnification of 20×, and the scale bar indicates 30 μm. The white arrows indicate F-actin alterations.

Similarly, in JB6 Cl41-5a cells (Figure 10) CHX 0.02% and 0.2% applied for 1, 2, and 3 min caused the highest modifications in F-actin structure (e.g., massive condensation) and cellular morphology (e.g., reduction in size, rounding, and shrinkage). A visible constriction of F-actin was also detected in this cell line after its exposure to CHX 0.0002%, for 3 min.

### 3.7. CHX 0.02% and 0.2% Induced an Apoptotic-like Nuclear Morphology

**Figure 11:**
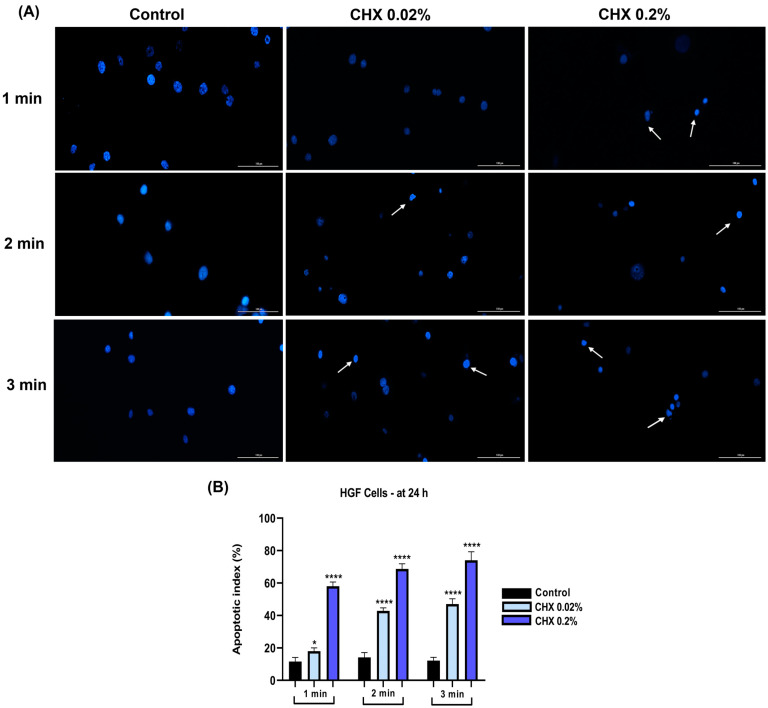
(**A**) Representative images showing the changes in nuclear morphology at 24 h following the 1, 2, and 3 min exposure of HGF cells to CHX 0.02 and 0.2%. The pictures were taken at a magnification of 20×, and the scale bar indicates 100 μm. The white arrows indicate nuclear modifications.(**B**) Analysis of the apoptotic index (%) in HGF cells at 24 h following the 1, 2, and 3 min exposure to CHX 0.02 and 0.2%. All data are expressed as mean values ± SD obtained from three independent experiments performed in triplicate. For analyzing the statistical differences between the control group and the CHX-stimulated groups, a one-way ANOVA test was conducted, followed by the Dunnet’s multiple comparison post-test; “*” indicates statistical significance (* *p* < 0.05; **** *p* < 0.0001).

Further, the influence of CHX on the nuclear aspect was evaluated. For this assay, the highest concentrations of CHX (0.02% and 0.2%) which caused the most significant cytotoxicity were evaluated. In HGF cells (Figure 11A), a reduction in the size of cell nuclei accompanied by condensation were evidenced (white arrows). The apoptotic index (AI) percentages increased with longer exposure and higher concentrations (Figure 11B).

**Figure 12:**
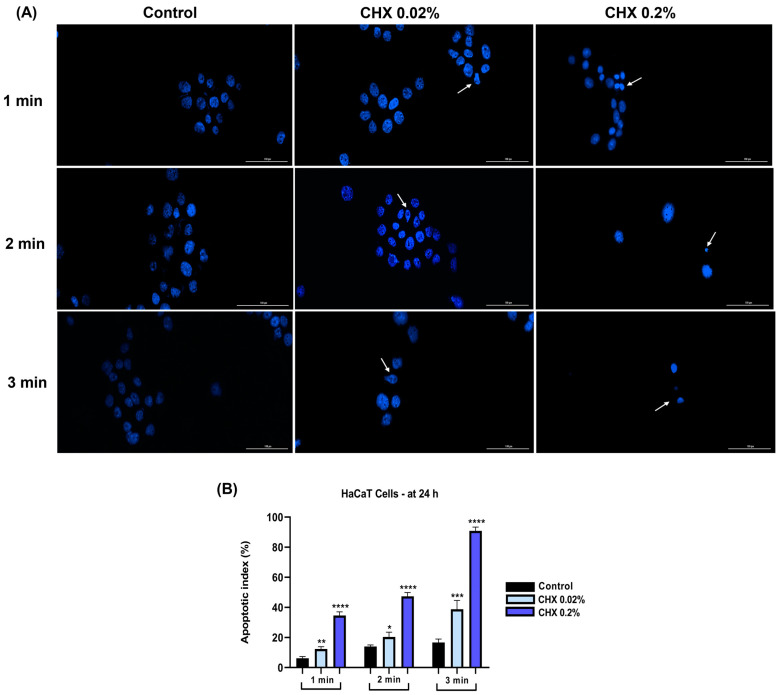
(**A**) Representative images showing the changes in nuclear morphology at 24 h following the 1, 2, and 3 min exposure of HaCaT cells to CHX 0.02 and 0.2%. The pictures were taken at a magnification of 20×, and the scale bar indicates 100 μm. The white arrows indicate nuclear modifications. (**B**) Analysis of the apoptotic index (%) of HaCaT cells at 24 h following the 1, 2, and 3 min exposure to CHX 0.02 and 0.2%. All data are expressed as mean values ± SD obtained from three independent experiments performed in triplicate. For analyzing the statistical differences between the control group and the CHX-stimulated groups, a one-way ANOVA test was conducted, followed by the Dunnet’s multiple comparison post-test; “*” indicates statistical significance (* *p* < 0.05; ** *p* < 0.01; *** *p* < 0.001; **** *p* < 0.0001).

In HaCaT cells (Figure 12A), CHX at the concentration of 0.02% caused nuclear dysmorphology, while a concentration of 0.2% induced a massive chromatin constriction. Regarding the apoptosis index (Figure 12B), it increased in a dose- and time-dependent manner, at the 0.2% concentration and the 3 min exposure time, the percentage reaching 90%.

**Figure 13:**
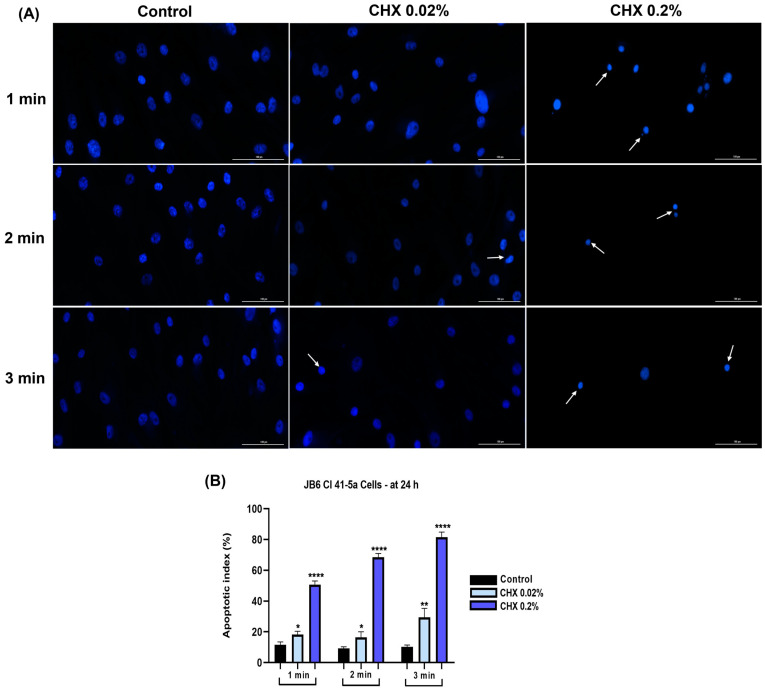
(**A**) Representative images showing the changes in nuclear morphology at 24 h following the 1, 2, and 3 min exposure of JB6 Cl 41-5a cells to CHX 0.02 and 0.2%. The pictures were taken at a magnification of 20×, and the scale bar indicates 100 μm. The white arrows indicate nuclear modifications. (**B**) Analysis of the apoptotic index (%) of JB6 Cl 41-5a cells at 24 h following the 1, 2, and 3 min exposure to CHX 0.02 and 0.2%. All data are expressed as mean values ± SD obtained from three independent experiments performed in triplicate. For analyzing the statistical differences between the control group and the CHX-stimulated groups, a one-way ANOVA test was conducted, followed by the Dunnet’s multiple comparison post-test; “*” indicates statistical significance (* *p* < 0.05; ** *p* < 0.01; **** *p* < 0.0001).

The nuclei of JB6 Cl 41-5a cells (Figure 13A) presented condensation, size reduction, fragmentation, and formation of apoptotic bodies; these signs were visible after their exposure to CHX 0.2% for 1, 2, and 3 min. The apoptotic index (Figure 13B) increased to approximately 80% compared to the control for the highest concentration tested, at the 3 min treatment.

### 3.8. CHX 0.02% and 0.2% Exerted an Irritant Effect on the Chorioallantoic Membrane

**Figure 14:**
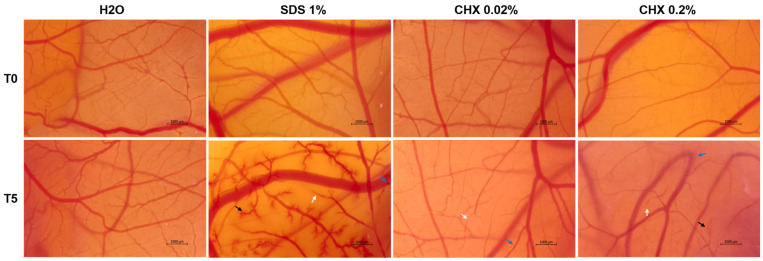
Representative images showing the aspect of the chorioallantoic membrane vasculature before treatment (T0) and 5 min after treatment with H_2_O (negative control), SDS 1% (positive control), CHX 0.02%, and CHX 0.2%. The scale bars indicate 1000 µm. The vascular changes are indicated using arrows (white—lysis; blue—coagulation; black—hemorrhage).

The potential irritant effect of CHX at two high concentrations (0.02% and 0.2%) was examined in ovo by means of the HET-CAM assay (Figure 14 and Table 2). Comparatively, H_2_O and SDS 1% were used as the negative and positive controls, respectively. According to the results, H_2_O caused no toxic events on the vasculature of the CAM, presenting a non-irritant effect, while SDS 1% caused hemorrhage, vascular lysis, and intravascular coagulation shortly after its application on the CAM, exerting a severe irritating potential. CHX at both tested concentrations showed an irritant action on the CAM; however, it was strong only at 0.2%. At 0.02%, CHX caused vascular lysis (white arrow) and coagulation (blue arrow) after the 5 min treatment, while no signs of hemorrhage were observed. At 0.2%, the lysis and coagulation caused by CHX were accompanied by microhemorrhages (black arrow).

## 4. Discussion

CHX stands as one of the most widely used antiseptic ingredients in dental products (i.e., mouthwashes, gels, toothpaste, etc.) due to its broad-spectrum antimicrobial activity and substantivity in the oral cavity which leads to a long-lasting and sustained effect [8]. Moreover, its clinical applications also extend to infection-preventing skin formulations used in hand antisepsis before surgery, preoperative baths, venepuncture, or catheter placement [1]. Although it is generally viewed as biocompatible [27], CHX use has been correlated with several adverse effects such as taste alteration, xerostomia, tongue or mouth pain, type I and IV hypersensitivity reactions, anaphylaxis, and wound healing prevention [2,16].

Thus, considering its numerous applications in clinical practice and potential toxic activities [28], the present research proposed a comprehensive preclinical evaluation of the biosafety profile of CHX by computationally predicting its toxic potential, while also assessing its cytopathic effect on a panel of healthy gingival and cutaneous cell lines and irritant activity on the CAM. The study was designed to investigate the deleterious effects of CHX at clinically relevant concentrations (0.0002%, 0.002%, 0.02%, and 0.2%) covering the doses at which it exerts bacteriostatic and bactericidal activities [29], and after a 24 h recovery period of the cells exposed to this antiseptic, knowing its ability to provide a long-lasting antimicrobial action, retain within the oral cavity, and bind to the stratum corneum of the skin [10,29].

First, the study resorted to the computational prediction of the toxic potential and drug-like features of CHX using the OSIRIS Property Explorer—an accurate and popular web tool that is useful in the forecast of physicochemical and toxicological drug properties through the estimation of drug-likeness, as well as of mutagenic, tumorigenic, irritant, and reproductive toxicity risks [12,30]. As shown in Table 1, CHX showed no predicted risk for any severe toxic effects and presented a negative drug likeness but a positive overall drug score.

The study then proceeded to the experimental cytotoxicity assessment of CHX on three established cell lines—HGF gingival fibroblasts, HaCaT immortalized keratinocytes, and JB6 Cl 41-5a epidermal cells—chosen as such to cover the clinical application of CHX on oral and cutaneous tissues, and due to their specific features. Gingival fibroblasts are elements of the periodontium playing a crucial role in tissue structure and integrity upkeep [31,32], and represent the most abundant cell type found within the oral cavity contributing to the maintenance of oral homeostasis. They were also previously employed in the safety evaluation of dental products [33]. Keratinocytes were selected due to their predominance within the layered squamous epithelium of the gingiva, and due to their previous application, along with fibroblasts, in skin toxicity studies [32,34]. In particular, HaCaT cells are very similar to isolated keratinocytes as regards their morphology, presence of major surface markers, and functionality, and were selected as models in cutaneous safety assessments, along with JB6 Cl 41-5a cells [35].

The obtained results indicated that the cytopathic effects of CHX were highly dependent on the tested concentration and exposure time. Therefore, according to Figure 1, Figure 2 and Figure 3, the most significant reductions in cell viability, number, and confluence were obtained after the treatment of all cell lines with CHX 0.02% and 0.2% for 3 min, following an incubation period of 24 h. We were also interested in exploring whether CHX harms the integrity of cell membranes by measuring the leakage of LDH in culture media (Figure 4). LDH is an enzyme present in all cells that is released within the extracellular environment following plasma membrane deterioration [36]. It was found that CHX disrupts cell membrane integrity only at the highest tested concentrations (0.02% and 0.2%), at all treatment times. Lower concentrations (0.0002% and 0.002%) had no considerable impact on LDH release from the cells’ cytoplasmic compartment. These results are in accordance with previous findings on the cytotoxicity of CHX salts. For instance, Pauline Vörös and co-workers previously showed that CHX digluconate 0.1% induced a cytotoxic effect on osteoblasts after 1, 5, and 10 min of stimulation, while also increasing LDH activity in the supernatant [37]. The study led by James X. Liu and his associates showed that CHX gluconate exerts a dose- and time-dependent cytotoxicity on primary human fibroblasts, osteoblasts, and myoblasts. At concentrations higher than 0.02%, a significant decrease in the percentage of viable cells was obtained at 48 h after their exposure to CHX for 1, 2, and 3 min [15]. Zhang M. et al. investigated the cytotoxicity of CHX acetate on HF and HaCaT cells; their results indicated a dose- and time-dependent decrease in cell viability, the greatest effect being reported after a 180 s exposure of both cell lines. In addition, they suggested that some slow-release formulations maintained a lasting tissue contact, with the prolonged exposure causing substantial cytotoxicity even at lower concentrations [38].

Next, the impact of CHX digluconate on cellular, cytoskeletal, and nuclear aspects was evaluated, considering that apoptosis can be distinguished from other cell death types (e.g., necrosis) through several morphological characteristics such as cell shrinkage, membrane blebbing, nuclear condensation, DNA fragmentation, apoptotic bodies formation, and cytoskeleton reorganization [39,40]. Several morphological impairments were observed in the cells treated with CHX digluconate, indicating its cytotoxic effect (Figure 5, Figure 6 and Figure 7). The shape changes were dependent on the cell type, CHX concentration, and exposure duration. Thus, cell elongation was evident in HGF cells treated with CHX 0.0002% and 0.002%, while shrinkage and reduction in size were observed at the highest concentrations (0.02% and 0.2%) after 1, 2, and 3 min of exposure. HaCaT cells showed a massive reduction in confluence, as well as shrinkage and rounding only after the treatment with CHX 0.02% and 0.2% at all time intervals; meanwhile, in the case of JB6 Cl 41-5a cells, massive cell destruction was noticed even at the lowest concentration, after the 3 min treatment. Lessa et al. observed a marked alteration in odontoblast-like cells’ morphology and a lower cell adherence after their exposure to CHX gluconate (0.06, 0.12, 0.2, 1, and 2%) for 60 s, 2 h, and 60 s with a 24 h recovery time; these events were more accentuated at higher CHX concentrations and treatment times [12]. CHX digluconate was also found to cause changes in osteoblast morphology such as shrinkage, inconsistent cell borders, detachment, enlargement, and reduced intercellular interaction [37].

As part of the cell morphology assessment, the impact of CHX treatment on actin distribution in HGF, HaCaT, and JB6 Cl 41-5a cells was also explored. Actin is the major protein constituent of the cytoskeleton [41], significantly contributing to numerous cell functions and processes such as cell mechanics, morphogenesis, membrane dynamics, proliferation, migration, and apoptotic cell death [40,42]. Additionally, actin also governs cell response to internal or external stimuli [43]. As presented in Figure 8, Figure 9 and Figure 10, CHX influenced the organization of F-actin bundles in HGF, HaCaT, and JB6 Cl 41-5a cells, leading to visible signs of dismorphology. The strongest effects illustrated by the massive condensation of F-actin bundles, cell rounding, shrinkage, and size reduction were caused by the treatment with the highest concentration of 0.2% at all the time intervals. In contrast to these results, a previous study demonstrated that the exposure of stem cells to CHX 0.12% for 30 s, 1.5 min, and 4.5 min caused no significant alterations within the actin cytoskeleton [44].

Nuclear shape deformation, constriction of chromatin, and fragmentation, as well as elevation of apoptotic index percentages, were observed in all three cell lines (Figure 11, Figure 12 and Figure 13), the most apoptotic-like nuclei being evident in the cells exposed to CHX at the highest concentration (0.2%). Giannelli et al. demonstrated that the 1 min treatment of fibroblasts, endothelial cells, and osteoblasts with CHX digluconate induced an apoptotic nuclear morphology [45].

Finally, the irritant potential of CHX digluconate 0.02% and 0.2% on the CAM developed in chicken fertilized eggs was assessed (Figure 14, Table 2). The HET-CAM method serves numerous applications in studies involving angiogenesis, inflammation, tissue engineering, drug delivery, and irritation assessment, addressing the potential mucosal use and local toxic reactions caused by a wide range of formulations [46]. We found that, despite the chemical-structure-based computational prediction (Table 1) indicating its lack of irritation risk, experimentally, CHX exerted an irritant effect on the CAM that was concentration-dependent. However, it was lower compared to SDS 1%, which was used as a positive control. A previous publication by Harnoss et al. revealed the CAM irritant effect of CHX digluconate applied for short-term, at a higher concentration (0.5%) than the ones tested in the present study [47].

Overall, the study provided a clinically relevant perspective on the delayed cytotoxicity of CHX on healthy gingiva- and skin-derived cell lines, representative of its topical applications in practice, at active antibacterial concentrations, and following a 24 h recovery time to mimic its tissue retention ability after direct contact. However, this study presents some limitations that should be addressed in future investigations. Firstly, the specific CHX concentration of 0.12% recommended before and after oral surgical interventions [27,48] was not evaluated herein; the current study aimed to extend the dose range as much as possible, from low, bacteriostatic concentrations to high, bactericidal concentrations. Secondly, the in vitro experiments were performed using representative 2D experimental models, while more advanced 3D models of the skin and the oral mucosa [49,50] could be further employed based on the findings related herein to complete the in vitro safety profile of CHX.

## 5. Conclusions

The current study illustrated that the exposure of healthy gingival and cutaneous cells to CHX digluconate results in concentration- and time-dependent cytotoxic events even after a 24 h period of recovery following treatment. This study thus unraveled the irritant effect of CHX on the chorioallantoic membrane, contributing to the portrayal of the overall toxicological profile of this broadly used antiseptic agent, and standing as a starting point for further safety investigations.

## Figures and Tables

**Table 1:** Computational prediction of the properties exerted by CHX by applying the OSIRIS Property Explorer Program.

CHX	MW	cLogP	Solubility	Drug-Likeness	Drug Score	Mutagenic, Tumorigenic, Irritant, and Reproductive Toxic Potential
	504.0	3.72	−7.92	−2.988	0.182	No risk

**Table 2:** Calculated irritation score (IS) for CHX 0.02% and CHX 0.2% using the HET-CAM assay. H_2_O was used as negative control, while SDS 1% represents positive control.

Sample	IS	Irritation Category
H_2_O	0.07	Non-irritant
SDS 1%	19.68	Severely Irritant
CHX 0.02%	8.55	Irritant
CHX 0.2%	11.96	Severely Irritant

## Data Availability

The data presented in this study are available on request from the corresponding author.
